# Relative abundance of total subgingival plaque-specific bacteria in salivary microbiota reflects the overall periodontal condition in patients with periodontitis

**DOI:** 10.1371/journal.pone.0174782

**Published:** 2017-04-03

**Authors:** Shinya Kageyama, Toru Takeshita, Mikari Asakawa, Yukie Shibata, Kenji Takeuchi, Wataru Yamanaka, Yoshihisa Yamashita

**Affiliations:** 1 Section of Preventive and Public Health Dentistry, Division of Oral Health, Growth and Development, Kyushu University Faculty of Dental Science, Fukuoka, Japan; 2 OBT Research Center, Kyushu University Faculty of Dental Science, Fukuoka, Japan; 3 YA Dental Clinic, Yonago, Japan; Tokyo Ika Shika Daigaku, JAPAN

## Abstract

Increasing attention is being focused on evaluating the salivary microbiota as a promising method for monitoring oral health; however, its bacterial composition greatly differs from that of dental plaque microbiota, which is a dominant etiologic factor of oral diseases. This study evaluated the relative abundance of subgingival plaque-specific bacteria in the salivary microbiota and examined a relationship between the abundance and severity of periodontal condition in patients with periodontitis. Four samples (subgingival and supragingival plaques, saliva, and tongue coating) per each subject were collected from 14 patients with a broad range of severity of periodontitis before periodontal therapy. The bacterial composition was analyzed by 16S rRNA gene amplicon sequencing using Ion PGM. Of the 66 species-level operational taxonomic units (OTUs) representing the mean relative abundance of ≥ 1% in any of the four niches, 12 OTUs corresponding to known periodontal pathogens, including *Porphyromonas gingivalis*, were characteristically predominant in the subgingival plaque and constituted 37.3 ± 22.9% of the microbiota. The total relative abundance of these OTUs occupied only 1.6 ± 1.2% of the salivary microbiota, but significantly correlated with the percentage of diseased sites (periodontal pocket depth ≥ 4 mm; r = 0.78, P < 0.001), in addition to the abundance of subgingival plaque microbiota (r = 0.61, P = 0.02). After periodontal therapy, the total relative abundance of these 12 OTUs was evaluated as well as before periodontal therapy and reductions of the abundance through periodontal therapy were strongly correlated in saliva and subgingival plaque (r = 0.81, P < 0.001). Based on these results, salivary microbiota might be a promising target for the evaluation of subgingival plaque-derived bacteria representing the present condition of periodontal health.

## Introduction

The human oral cavity harbors numerous, diverse, indigenous microorganisms. They create distinct microbial communities on intraoral surfaces, such as the tooth surface, gingival crevice, tongue dorsum, and buccal mucosa, with different ecological conditions. Subgingival microbiota is comprised of a unique microbial community dominated by obligatory anaerobic and proteolytic bacteria, which are involved in the progression of periodontitis, one of the major causes of tooth loss. *Porphyromonas gingivalis*, *Tannerella forsythia*, and *Treponema denticola* are shown to be strongly associated with diseased periodontal pocket and are known as red complex, which has been the focus as the etiologic agents of periodontitis [1]. Furthermore, based on the results of the open-ended 16S rRNA gene analyses, a recent systematic review added 17 bacterial taxa to the list of suspected periodontal pathobionts [2, 3].

Saliva is constantly secreted from the salivary glands into the oral cavity and contains diverse bacteria shed from various oral sites. The periodontal pathobionts detached from subgingival microbiota are also identified in saliva [4–8]. Their presence or absence was reported to be associated with periodontal status [9]. Saliva can be collected easily, repetitively, and noninvasively. Therefore, sampling the salivary microbiota seems to be a promising way to diagnose periodontal conditions. On the other hand, several studies have demonstrated that the bacterial composition in saliva differs greatly from that of dental plaque, including both supra- and subgingival microbiota, and it is closer to that on the mucosal surfaces, such as the tongue coating [10–13]. Our previous study also showed a distinction between the community structure of microbiota between supragingival plaque and saliva, as well as, the compositional stability of the salivary microbiota against a supragingival microbiota shift [14]. These facts invoke doubt about the clinical utility of a comprehensive analysis of salivary microbiota for monitoring subgingival microbiota to evaluate periodontal conditions.

In this study, we focused on subgingival plaque-specific bacteria that were particularly predominant in subgingival plaque compared to those in other oral niches. We assumed that the relative abundance of subgingival plaque-specific bacteria in saliva would be useful to monitor the overall periodontal condition easily and noninvasively, regardless of heterogeneous clinical conditions. Therefore, we collected samples of four different niches (subgingival and supragingival plaques, saliva, and tongue coating) in mouths of 14 patients diagnosed with chronic periodontitis with heterogeneous clinical conditions (percentage of diseased sites, age, sex, the number of remaining tooth, etc.) before and after the periodontal treatment. The microbiota composition of each sample was determined by using 16S rRNA gene amplicon deep sequencing analyses and subgingival plaque-specific bacteria were identified. Then we revealed a good correlation between periodontal conditions and the relative abundances of the subgingival plaque-specific bacteria in saliva, and the synchronizing shift of their relative abundance in subgingival plaque and saliva following periodontal therapy. Here we emphasize the clinical utility of evaluating the relative abundances of the subgingival plaque-specific bacteria in saliva for screening periodontal health condition in whole based on the findings in the present study.

## Materials and methods

### Ethics statement

All subjects understood the nature of the study and provided written informed consent. The ethics committee of Kyushu University Faculty of Dental Science approved this study and the procedure for obtaining informed consent (approval number, 19B-3).

### Study population and sample collection

The subjects consisted of 14 patients who visited the YA Dental Clinic in Yonago, Tottori, Japan, and were diagnosed with chronic periodontitis. They were a subgroup analyzed in our previous study [14]. The subjects who used antibiotics or underwent periodontal surgery in the 6 months preceding this study have been excluded. Five out of 19 subjects in previous study were excluded in this study because not all of four samples (subgingival and supragingival plaques, saliva, and tongue coating) were sufficient for PCR amplification required for the 16S rRNA gene amplicon sequencing.

Sample collections and clinical examinations were conducted at the initial visit and after periodontal therapy (approximately 2 years later), as described previously [14]. The subjects were asked to chew gum for 5.5 min and stimulated saliva samples were collected in sterile plastic tubes during the final 5 min. Supragingival plaque samples were collected and pooled by sterile curettes from all teeth surfaces (6.5 ± 0.9 teeth), on the side of the upper half-jaw that contained the most teeth. Subgingival plaque samples were collected and pooled from gingival crevices of the same region by sterile curettes. Tongue coating samples were collected by scraping the tongue with a sterile plastic spatula from the dorsum of the tongue. The periodontal pocket depths and bleeding on probing (BOP) at six sites (mesiobuccal, midbuccal, distobuccal, mesiolingual, midlingual, and distolingual) of all teeth were measured using a periodontal pocket probe following sample collection. Oral hygiene status was assessed by the plaque control record (PCR) [15]. DNA was extracted from each sample using the beads-beating method [14] and stored at -30°C until further analysis.

### Ion Torrent 16S rRNA gene amplicon sequencing analysis

A total of 112 samples (four each from 14 patients in pre- and post-periodontal therapy) were examined using barcoded pyrosequencing analysis of the 16S rRNA gene using Ion PGM (Thermo Fisher Scientific, MA, USA), a next-generation sequencer. The V1–V2 regions of 16S rRNA genes from each sample were amplified using the following primers: 8F (5’-AGA GTT TGA TYM TGG CTC AG-3’) with the Ion Torrent adaptor A and the sample-specific 8-base tag sequence and 338R (5’-TGC TGC CTC CCG TAG GAG T-3’) with the Ion Torrent trP1 adaptor sequence. PCR amplification, purification, and quantification of each PCR amplicon was performed as previously described [16]. Equal amounts of the purified PCR amplicons were pooled together and gel-purification was accomplished using Wizard SV Gel and PCR Clean-Up System (Promega, WI, USA). The DNA concentration was determined using a KAPA Library Quantification Kit (KAPA Biosystems, MA, USA) and the DNA was diluted to 8 pM for use as the template DNA for emulsion PCR. Emulsion PCR and enrichment of template-positive particles were performed using an Ion PGM Template OT2 400 Kit (Thermo Fisher Scientific) in Ion One Touch 2 system (Thermo Fisher Scientific). The enriched particle was loaded onto an Ion 318 v2 chip (Thermo Fisher Scientific) and sequencing was performed on the Ion PGM (Thermo Fisher Scientific) using an Ion PGM Hi-Q Sequencing kit (Thermo Fisher Scientific).

### Data analysis and taxonomy assignment

Quality filtering of raw sequence reads was performed using a script written in R (version 3.1.1). The reads were excluded from the analysis if they were ≤ 200 bases (not including the tag sequence), had an average quality score ≤ 25, did not include the correct forward primer sequence, did not include the correct reverse primer sequence (one mismatch was allowed), or had a homopolymer run > 7 nt. The quality-checked reads were assigned to the appropriate sample by examining the tag sequence. Similar sequences were assigned into operational taxonomic units (OTUs) using UPARSE [17] as described previously [16], with a minimum pairwise identity of 96%. The taxonomy of representative sequences was determined using BLAST against 831 oral bacterial 16S rRNA gene sequences (HOMD 16S rRNA RefSeq version 13.2) in the Human Oral Microbiome Database [18]. Nearest-neighbor species with ≥ 98% identity were selected as candidates for each representative OTU. The taxonomy of sequences without hits was further determined using RDP classifier with a minimum support threshold of 80%. Unifrac analysis [19], following rarefaction to 4,000 reads per sample, was performed using QIIME [20] as described previously [16]. The number of OTUs were calculated following rarefaction to 4,000 reads per sample using R. A hierarchical cluster analysis of dominant OTUs was conducted based on the Bray-Curtis index using R.

### Statistical analysis

All statistical analyses were performed using R (version 3.1.1). A Pearson correlation test was used to evaluate the relationship between the relative abundance or relative abundance shift of each bacterial species in different samples and between the relative abundance of each bacteria and the periodontal condition of each subject.

## Results

Subgingival and supragingival plaques, saliva, and tongue coating samples (their microbiota were defined as SUBP, SUPP, SL, and TC, respectively) were collected twice from 14 patients with periodontitis who visited a dental clinic (5 women and 9 men; aged 35–73 years) at their initial visit (pre-therapy samples) and after periodontal therapy (approximately 2 years later; post-therapy samples). The subjects had completed periodontal therapy and received supportive therapy with maintenance care during the interval between the sample collections. Their clinical periodontal conditions improved from that during the initial sample collection (S1 Table).

The bacterial composition of each sample was determined by 16S rRNA gene amplicon analysis. Of the 2,882,175 reads obtained, 2,081,029 high-quality reads were used in the analysis. The sequences were assigned to 399 species-level OTUs using a cut-off distance of 0.04. The similarity between the overall microbiota compositions of the pre-therapy samples obtained from each niche was assessed using weighted UniFrac distance metric. A principal coordinate analysis (PCoA) plot demonstrated that SL was distinct from both SUBP and SUPP, but closely similar to TC (S1 Fig). Whereas the difference was observed between SUPP and SUBP, interestingly SUPP was closer to SL than SUBP in the PCoA plot. In both SL and TC, *Streptococcus* was the most predominant and *Prevotella*, *Veillonella*, *Rothia*, and *Actinomyces* were present in higher proportions (S2 Fig). On the other hand, *Fusobacterium*, *Leptotrichia*, *Porphyromonas*, *Corynebacterium*, and *Capnocytophaga* were found in significantly higher proportions in both SUBP and SUPP. The SUBP was distinguished from SUPP by a relatively higher abundance of *Porphyromonas* and *Fusobacterium*.

**Fig 1:**
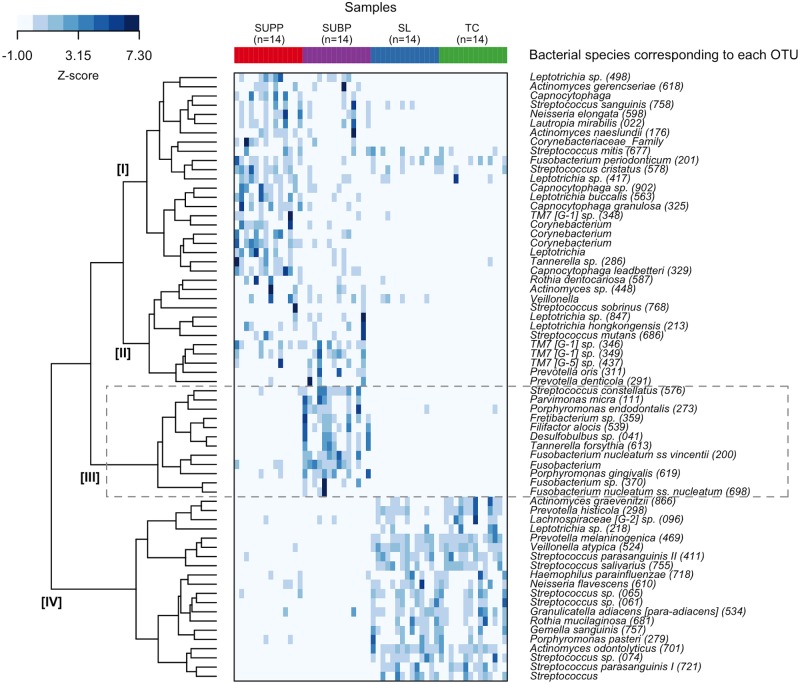
Relative abundance distribution of the 66 OTUs whose mean relative abundances in the pre-therapy samples from any of 4 niches exceeded 1%. The relative abundances of each OTU were normalized to a mean of 0 with standard deviation of 1 (z-score normalization) and are represented by the blue gradient in each grid (light = low abundance; dark = high abundance). The OTUs were ordered based on the result of a hierarchical cluster analysis using the Bray-Curtis distance, which is depicted as a dendrogram on the left side of the diagram. The 12 OTUs belonging to cluster III were characteristically more predominant in SUBP as compared to microbiota from the other three niches. It is displayed in the box with a broken line. Oral taxon IDs were given in parentheses following bacterial names.

Of the 399 species-level OTUs identified in this study, the mean relative abundances of the 66 OTUs exceeded 1% in the pre-therapy samples from any of 4 niches. The relative abundances of these dominant OTUs in each sample were displayed as a heatmap (Fig 1). The results of a hierarchical cluster analysis, which is shown as a dendrogram in the left side of the diagram, suggested the stratification of these OTU into four groups (Cluster I, II, III, and IV). The 12 OTUs belonging to cluster III were characteristically predominant in SUBP compared to the samples from the other 3 niches and these 12 OTUs were designated SUBP-specific OTUs. Most of these OTUs were assigned to periodontitis-associated bacteria, including *P*. *gingivalis* and *T*. *forsythia*.

**Fig 2:**
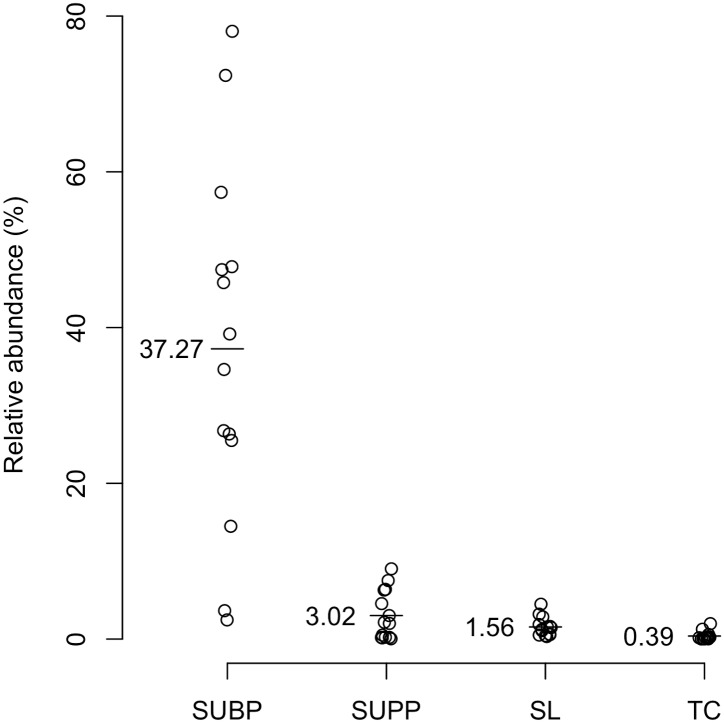
Total relative abundances of the 12 SUBP-specific OTUs in the pre-therapy samples from each niche. The mean relative abundance in each niche is represented by a line and a number.

**Fig 3:**
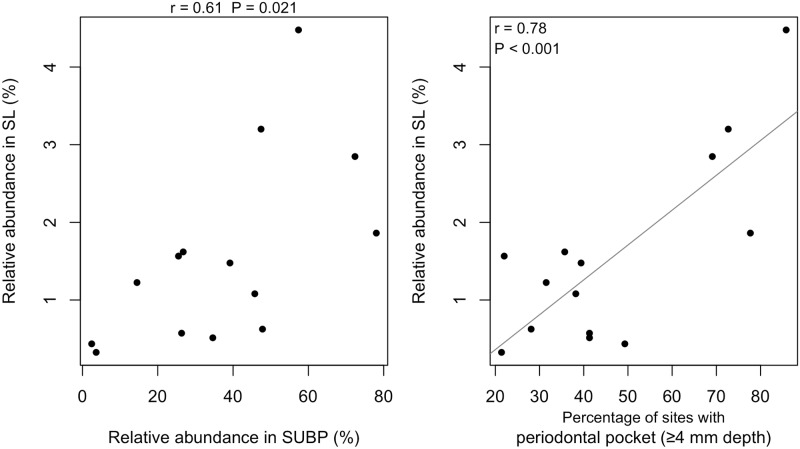
Correlation of the total relative abundance of the 12 SUBP-specific OTUs in SL and SUBP samples, or the percentage of sites with periodontal pockets (≥4 mm depth). The Pearson correlation coefficient (r) and the P value are shown in the upper or upper left side of the diagram. The gray line depicts the regression line.

The 12 OTUs comprised of 37.3 ± 22.9% of SUBP and their relative abundance in SUBP differed among the subjects in this study (2.5% to 78.0%; Fig 2) and correlated with the percentage of diseased sites (periodontal pocket depth ≥ 4 mm; Pearson’s correlation coefficient, r = 0.70, P < 0.01; S3 Fig). These species seem to preferably inhabit diseased sites in the deep gingival crevices. They were also identified in SUPP, SL, and TC, but in the minority of the microbiota (3.0 ± 3.2%, 1.6 ± 1.2% and 0.3 ± 0.6%, respectively; Fig 2). Their relative abundance in SUBP significantly correlated with those in SL and TC (r = 0.61 and 0.53, P = 0.021 and 0.049, respectively; Fig 3, S4 Fig), but not that in SUPP. The significant correlation between the relative abundance of these OTUs in SL and the clinical periodontal condition was also observed (r = 0.78, P < 0.001; Fig 3). Moreover, it is interesting to know the highest correlation coefficient was found in the total relative abundances of 12 SUBP-specific OTUs in SL when compared to those of any OTU among the 12 OTUs (Table 1).

**Table 1:** Correlation of the relative abundance of each SUBP-specific OTU in SL and the percentage of sites with periodontal pockets (≥4 mm depth).

Bacterial species corresponding to each OTU	r	P value
*Streptococcus constellatus* (576)	0.416	0.139
*Parvimonas micra* (111)	0.448	0.108
*Porphyromonas endodontalis* (273)	0.703	0.005
*Fretibacterium* sp. (359)	0.525	0.054
*Filifactor alocis* (539)	0.572	0.033
*Desulfobulbus* sp. (041)	0.559	0.038
*Tannerella forsythia* (613)	0.700	0.005
*Fusobacterium nucleatum* ss *vincentii* (200)	0.267	0.356
*Fusobacterium*	0.241	0.407
*Porphyromonas gingivalis* (619)	0.381	0.179
*Fusobacterium* sp. (370)	0.006	0.984
*Fusobacterium nucleatum* ss. *nucleatum* (698)	0.358	0.209
Total of the 12 OTUs	0.784	<0.001

Oral taxon IDs were given in parentheses following bacterial names.

**Fig 4:**
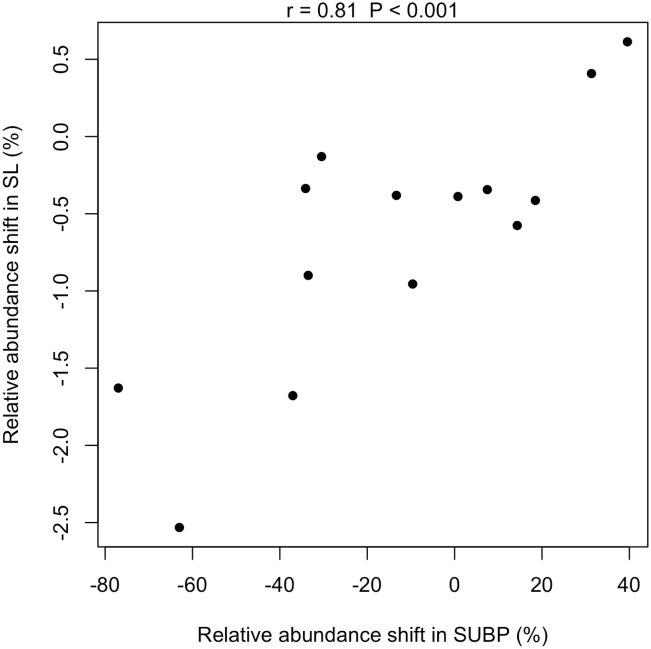
Correlation of total relative abundance shift of the 12 SUBP-specific OTUs in SUBP samples with that in SL samples following periodontal therapy. The Pearson correlation coefficient (r) and the P value are shown in the upper side of the diagram.

The relative abundances of the 12 OTUs decreased in both SL and SUBP after periodontal therapy, followed by the improvement of periodontal condition. The shift in their abundance in SL was strongly correlated with that in SUBP (r = 0.81, P < 0.001; Fig 4). In addition, their shift in abundance in SUBP, as well as that in SL, was also significantly associated with a decrease in the percentage of diseased sites following periodontal therapy (r = 0.62, P = 0.02 in SUBP; r = 0.58, P = 0.03 in SL; S5 Fig).

## Discussion

This study identified 12 species-level OTUs that were characteristically more predominant in SUBP than in the other 3 microbiota (SUPP, SL and TC) and demonstrated that their relative abundances and shifts in relative abundance in SUBP and SL were strongly correlated with periodontal health. Our results showed that the composition of the SL was more similar to that of TC than that of SUBP, which is consistent to the findings of previous reports [10–13]. In addition, considering that these 12 OTUs made up 37.3 ± 22.9% of the SUBP, but accounted for only 1.6 ± 1.2% of the SL (Fig 2), bacteria derived from periodontal pockets are a minority assemblage of SL containing bacteria shed from various oral sites. Nevertheless, the present results showed that the relative abundance of the SUBP-specific OTUs in SL monitored by using a 16S rRNA gene deep sequencing approach can reflect that in SUBP, representing the condition of periodontal health.

Most of the 12 SUBP-specific OTUs corresponded to well-known periodontitis-associated bacteria (Fig 1), such as the “red complex” bacteria (*P*. *gingivalis* and *T*. *forsythia)* and “orange complex” bacteria (*Fusobacterium nucleatum*, *Parvimonas micra*, and *Streptococcus constellatus)*, and were observed in higher proportions in subgingival plaque at the deeper periodontal pockets [1]. *Porphyromonas endodontalis*, *Filifactor alosis*, *Desulfobulbus* sp. HOT-041, and *Fretibacterium* sp. HOT-359 were also newly implicated in periodontitis by multiple recent studies using the comprehensive molecular approach [2]. One of 12 OTUs did not correspond to any of oral bacterial sequences deposited in the Human Oral Microbiome Database [18]. Its nucleotide sequence was assigned to genus *Fusobacterium* and it exhibited 97.6% identity with *Fusobacterium nucleatum* OT-420. It is reasonable to consider that their predominance in SL would indicate high periodontopathogenic activity in SUBP. In fact, their relative abundances in SL were significantly correlated with the severity of periodontitis (Fig 3) and their shift in relative abundance following periodontal therapy was significantly associated with a decrease of the diseased sites in this study (S5 Fig). These results suggest that surveillance of SUBP-specific bacteria via saliva would be useful as an indicator of periodontal health.

The alternative use of SL for monitoring SUBP has been classically investigated. Umeda *et al*. demonstrated that the presence or absence of periodontal pathogens, including *P*. *gingivalis*, in SL were associated with that in SUBP by using a PCR detection-based method [21]. It was also confirmed by later studies using more sophisticated methods, such as quantitative PCR [22] and microarray [23]. Our current results derived by a 16S rRNA gene sequencing approach using a next-generation sequencer contributed further evidence that the analysis of SL provides precise information on the relative difference in abundance and shift of SL. It is noteworthy that the total relative abundance of the 12 SUBP-specific bacteria in SL correlated better with periodontal health than the relative abundance of any of the 12 SUBP-specific bacteria (Table 1). This data suggests that the detection of multiple bacterial species in SL is superior to the detection of a sole bacterial species when evaluating the progression of a mixed infection, such as periodontal disease.

Our previous study indicated that SUPP had little effect on the composition of SL [14]. In the present study, the relative abundance shifts before and after periodontal therapy of SUPP-specific OTUs (22 OTUs classified into cluster I in Fig 1) in SUPP did not significantly correlate with that in SL (S6 Fig), even though the relative abundance of SUPP-specific OTUs in SL was significantly associated with that in SUPP prior to periodontal therapy (S7 Fig). However, in contrast to the SUBP-specific bacteria, SUPP-specific OTUs occupied nearly 10% of the microbiota in every niche (S8 Fig) and they were not very unique to SUPP, suggesting that SUPP-specific OTUs in sliva would be less helpful for evaluating the condition of the SUPP.

Prior to periodontal therapy, all subjects had a periodontal pocket depth ≥ 4 mm with broad range of percent sites from 21.4% to 85.8%. It remains unclear whether a significant correlation between the relative abundance of the SUBP-specific bacteria in SL and periodontal health condition is observed within only subjects healthier than our subjects. Additional research with a larger number of subjects, including healthy subjects, is needed to confirm the clinical value of evaluating SUBP-specific bacteria for diagnosing periodontal conditions. However, the relative abundance of SUBP-specific bacteria in SL generally decreased after periodontal therapy exhibiting a significance association with clinical improvement (S5 Fig), suggesting that the relative abundance of SUBP-specific bacteria in SL is promising as a clinical assessment tool for periodontal health.

The relative abundances of dominant bacterial genera in SL were highly similar to those in TC (S2 Fig). TC-specific OTUs could not be discriminated from SL-specific OTUs in the cluster analysis (Fig 1). This result is consistent with previous evidence that TC is the dominant source of the bacterial population in SL [10–13]. However, the identification of more OTUs from SL than TC in all subjects indicates that SL also contains bacteria shed from other oral niches, including periodontal pocket, in addition to tongue dorsum (S9 Fig).

In this study, the sample size was small (14 patients) because of inconveniences and difficulty with sample collection, especially with the two plaque samples from all tooth surfaces in the upper half-jaw. We identified 12 OTUs as SUBP-specific bacteria in the present study, but we are not able to exclude the possibility that additional species might be suitable for monitoring periodontal conditions. For example, *Treponema denticola* was not selected as a SUBP-specific bacteria because the relative abundance was slightly less than 1%, but it was strictly specific to SUBP. In addition, clinical attachment loss of each individual was not assessed in this study. Further study with a larger sample size and evaluating other relevant parameters of periodontal health would be needed to determine SUBP-specific bacteria and verify the present results.

There is a possibility that the combination of SUBP-specific bacteria affects the prognosis of periodontal diseases. Indeed, not all of the SUBP-specific bacteria in SUBP increased with periodontal severity and the relative abundance of each of SUBP-specific bacteria in SL did not necessarily correlate with the periodontal condition. Therefore, a comprehensive bacteriological examination should be considered in order to explore the key finding in the relationship between bacterial combination and periodontal prognosis. However, there are usually too many sites of periodontal pockets to be explored by bacteriological examination. Evaluation of SUBP-specific bacteria in saliva might be a promising bacteriological examination in the future of periodontal therapy, substituting for the cost consuming bacteriological examinations at every diseased site.

## Supporting information

S1 Fig A principal coordinate analysis plot showing the similarity between pre-therapy samples from four oral niches. Plots were generated using weighted UniFrac distance metric. Samples collected from 4 oral niches are depicted using different colors. These two components explain the 55.1% variance.
(TIFF)

S2 Fig Mean relative abundances of bacterial genera in pre-therapy samples from each niche. Only 11 genera with a mean relative abundance of ≥ 5% within each of the 4 niches are shown.
(TIFF)

S3 Fig Correlation of the total relative abundance of the 12 subgingival plaque-specific Operational Taxonomic Units (OTUs) in Subgingival Plaque (SUBP) samples with the percentage of sites with periodontal pockets (≥4 mm depth). The Pearson correlation coefficient (r) and the P value are shown in the upper left side of the diagram. The gray line represents the regression line.
(TIFF)

S4 Fig Correlation of the total relative abundance of the 12 subgingival plaque-specific Operational Taxonomic Units (OTUs) in Subgingival (SUBP) and Supragingival (SUPP) plaque samples, or Tongue Coating (TC) samples. The Pearson correlation coefficient (r) and the P value are shown in the upper side of the diagram.
(TIFF)

S5 Fig Correlation of the total relative abundance shift of the 12 subgingival plaque-specific Operational Taxonomic Units (OTUs) following periodontal therapy in Subgingival Plaque (SUBP) and Saliva (SL) samples and the transition of percentage of sites with periodontal pockets (≥4 mm depth). The Pearson correlation coefficient (r) and the P value are described in the upper left side of the diagram. The gray line depicts the regression line.
(TIFF)

S6 Fig Correlation of total relative abundance shift of the 22 supragingival plaque-specific Operational Taxonomic Units (OTUs) following periodontal therapy in Supragingival Plaque (SUPP) samples and that in Saliva (SL) samples. The Pearson correlation coefficient (r) and the P value are shown in the upper side of the diagram.
(TIFF)

S7 Fig Correlation of the total relative abundance of the 22 supragingival plaque-specific Operational Taxonomic Units (OTUs) in Supragingival Plaque (SUPP) samples with that in Saliva (SL) samples. (TIFF)

S8 Fig Total relative abundances of the 22 OTUs that are characteristically predominant in supragingival plaque samples in the pre-therapy samples from each niche. The mean relative abundance in each niche is represented by a line and a number.
(TIFF)

S9 Fig The number of identified Operational Taxonomic Units (OTUs) in each Saliva (SL) and Tongue Coating (TC) sample. The number of OTUs is calculated following rarefaction to 4,000 reads per sample using R.
(TIFF)

S1 Table Clinical condition of each subject in this study. BOP, bleeding on probing; PCR, plaque control record.
(DOCX)
