# Supplementary figures and images for: Relative abundance of total subgingival plaque-specific bacteria in salivary microbiota reflects the overall periodontal condition in patients with periodontitis
Source: PLoS One. 2017 Apr 3;12(4):e0174782. doi: 10.1371/journal.pone.0174782 (PMC5378373; doi:10.1371/journal.pone.0174782)

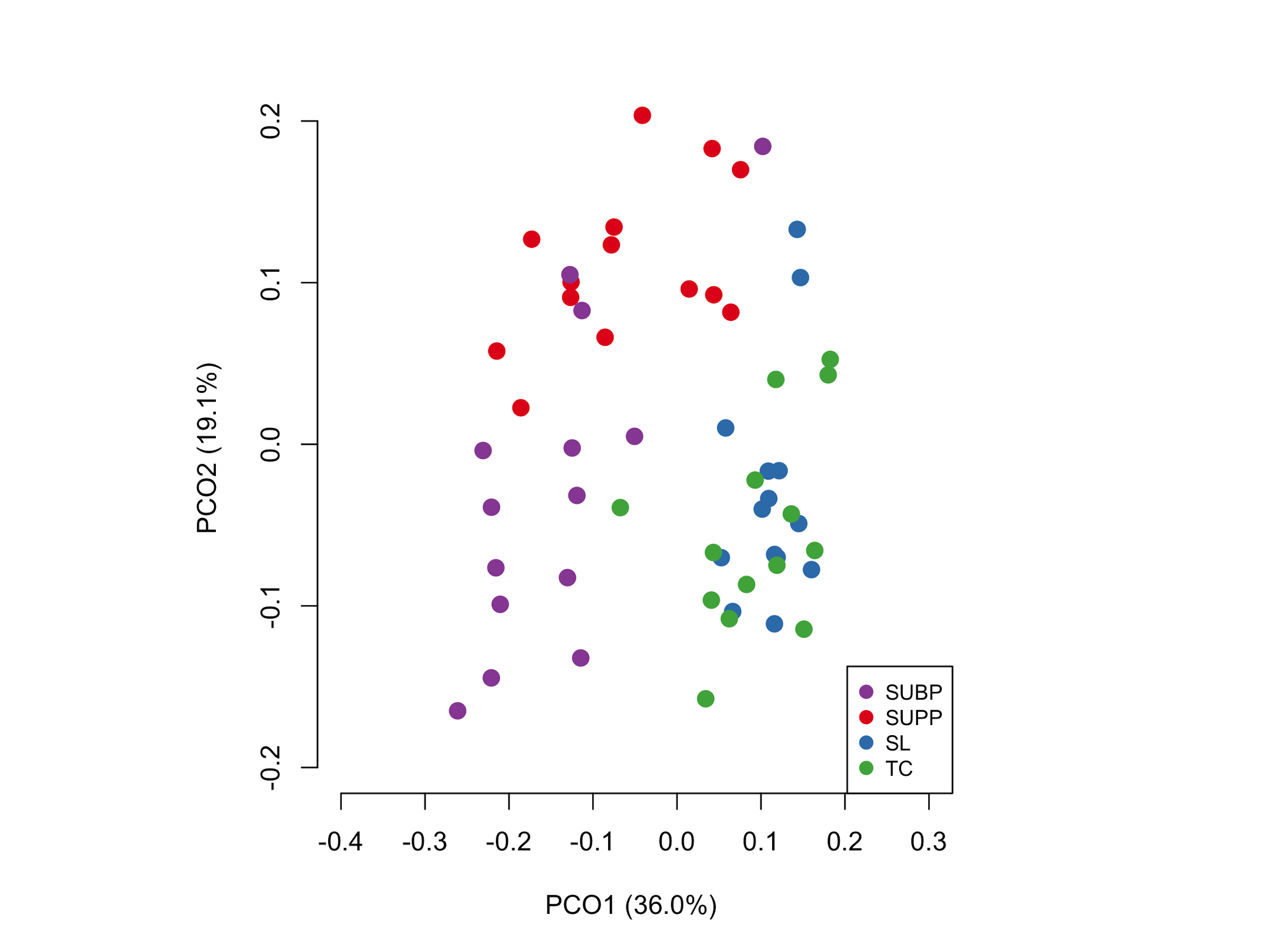

Supplement: S1 Fig — Plots were generated using weighted UniFrac distance metric. Samples collected from 4 oral niches are depicted using different colors. These two components explain the 55.1% variance. (TIFF) [file pone.0174782.s001.tiff]

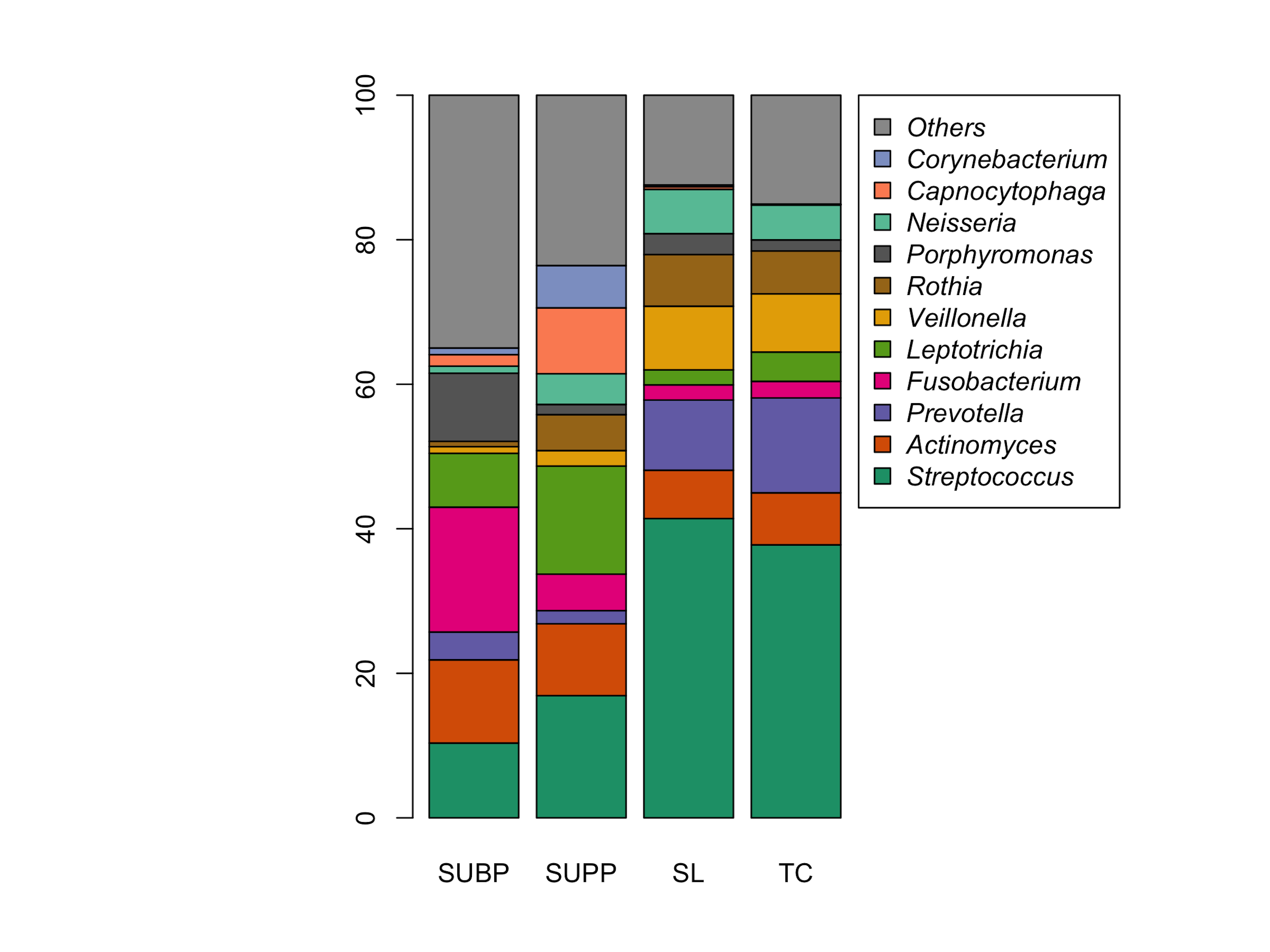

Supplement: S2 Fig — Only 11 genera with a mean relative abundance of ≥ 5% within each of the 4 niches are shown. (TIFF) [file pone.0174782.s002.tiff]

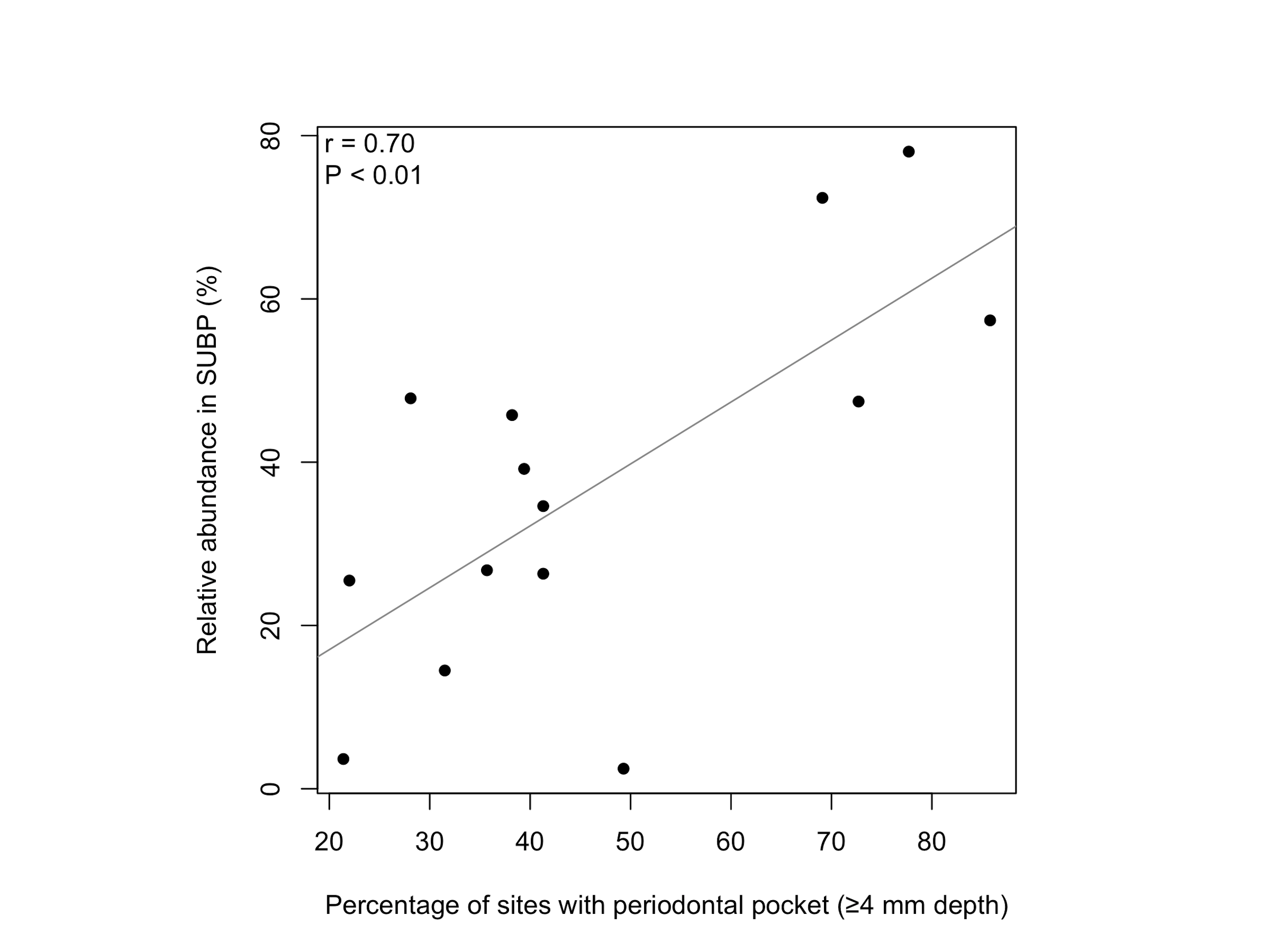

Supplement: S3 Fig — The Pearson correlation coefficient (r) and the P value are shown in the upper left side of the diagram. The gray line represents the regression line. (TIFF) [file pone.0174782.s003.tiff]

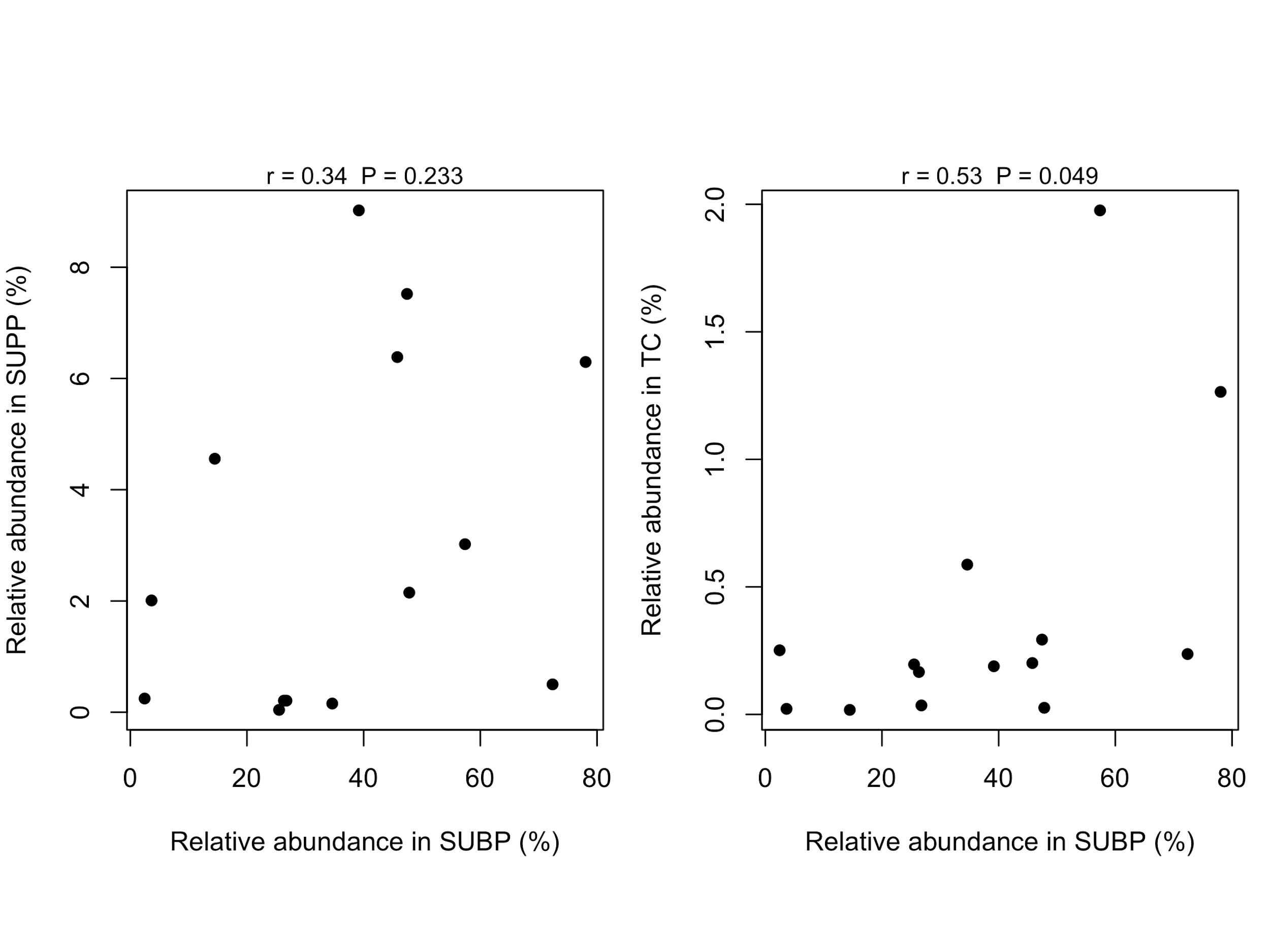

Supplement: S4 Fig — The Pearson correlation coefficient (r) and the P value are shown in the upper side of the diagram. (TIFF) [file pone.0174782.s004.tiff]

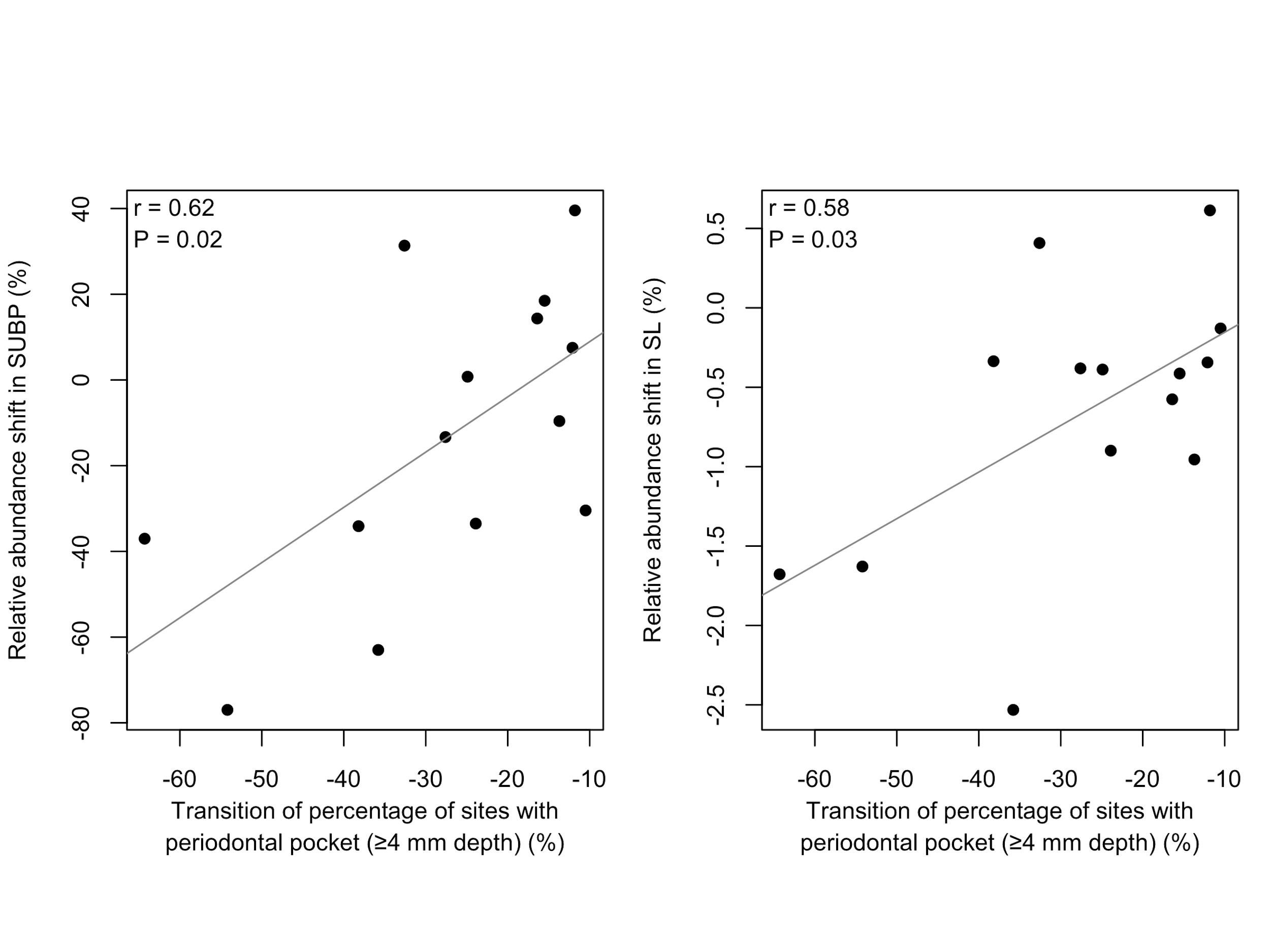

Supplement: S5 Fig — The Pearson correlation coefficient (r) and the P value are described in the upper left side of the diagram. The gray line depicts the regression line. (TIFF) [file pone.0174782.s005.tiff]

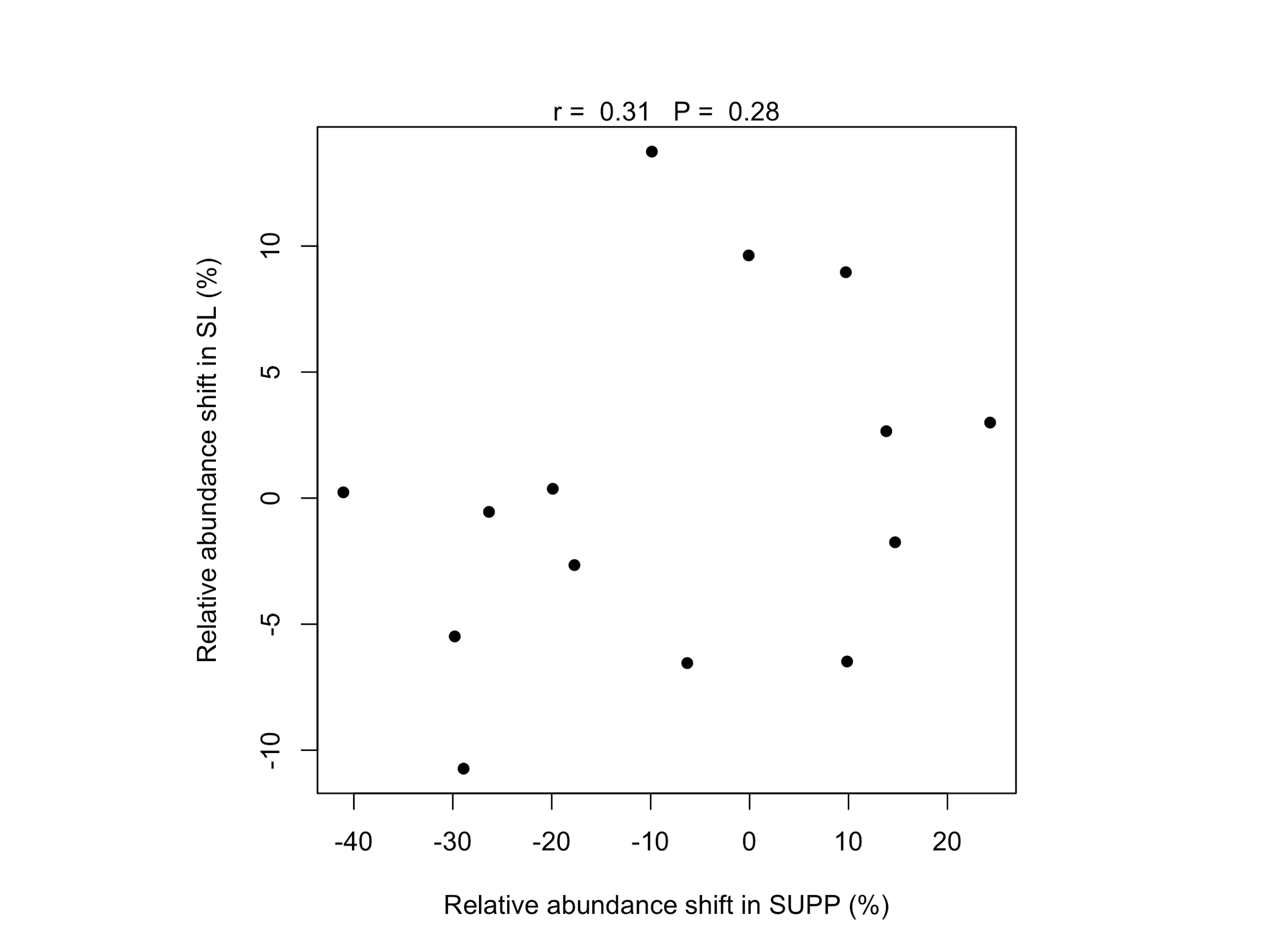

Supplement: S6 Fig — The Pearson correlation coefficient (r) and the P value are shown in the upper side of the diagram. (TIFF) [file pone.0174782.s006.tiff]

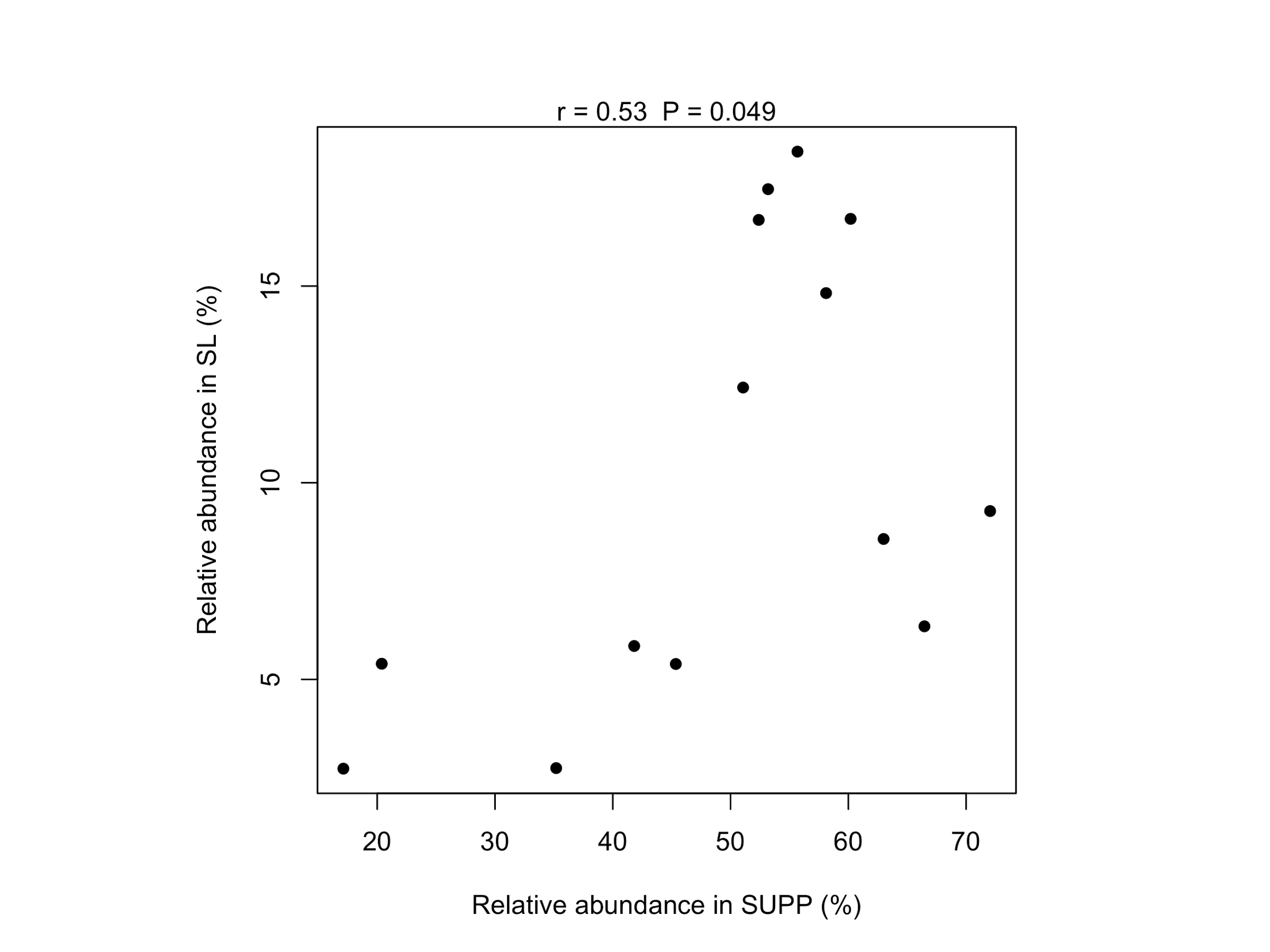

Supplement: S7 Fig — (TIFF) [file pone.0174782.s007.tiff]

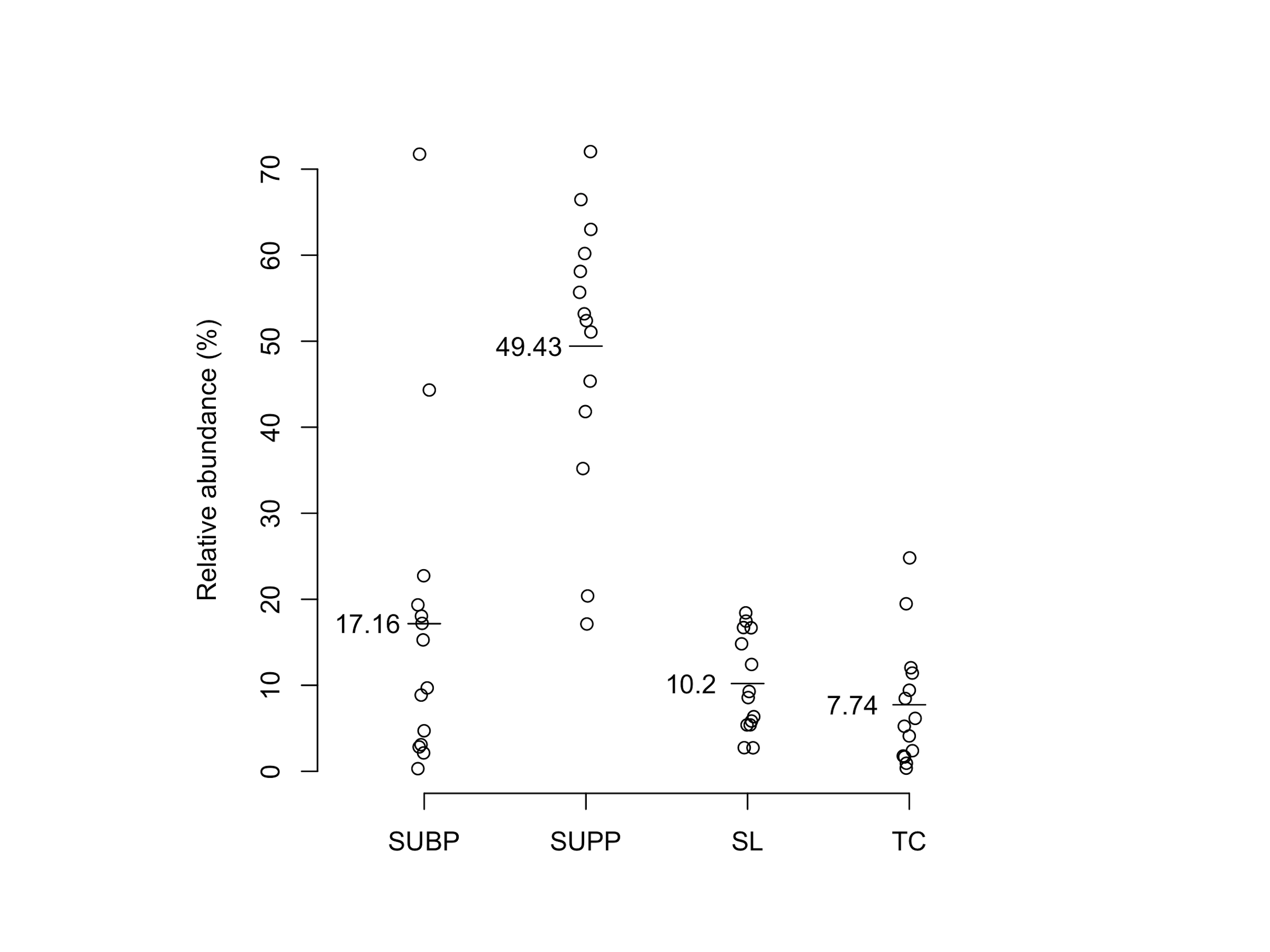

Supplement: S8 Fig — The mean relative abundance in each niche is represented by a line and a number. (TIFF) [file pone.0174782.s008.tiff]

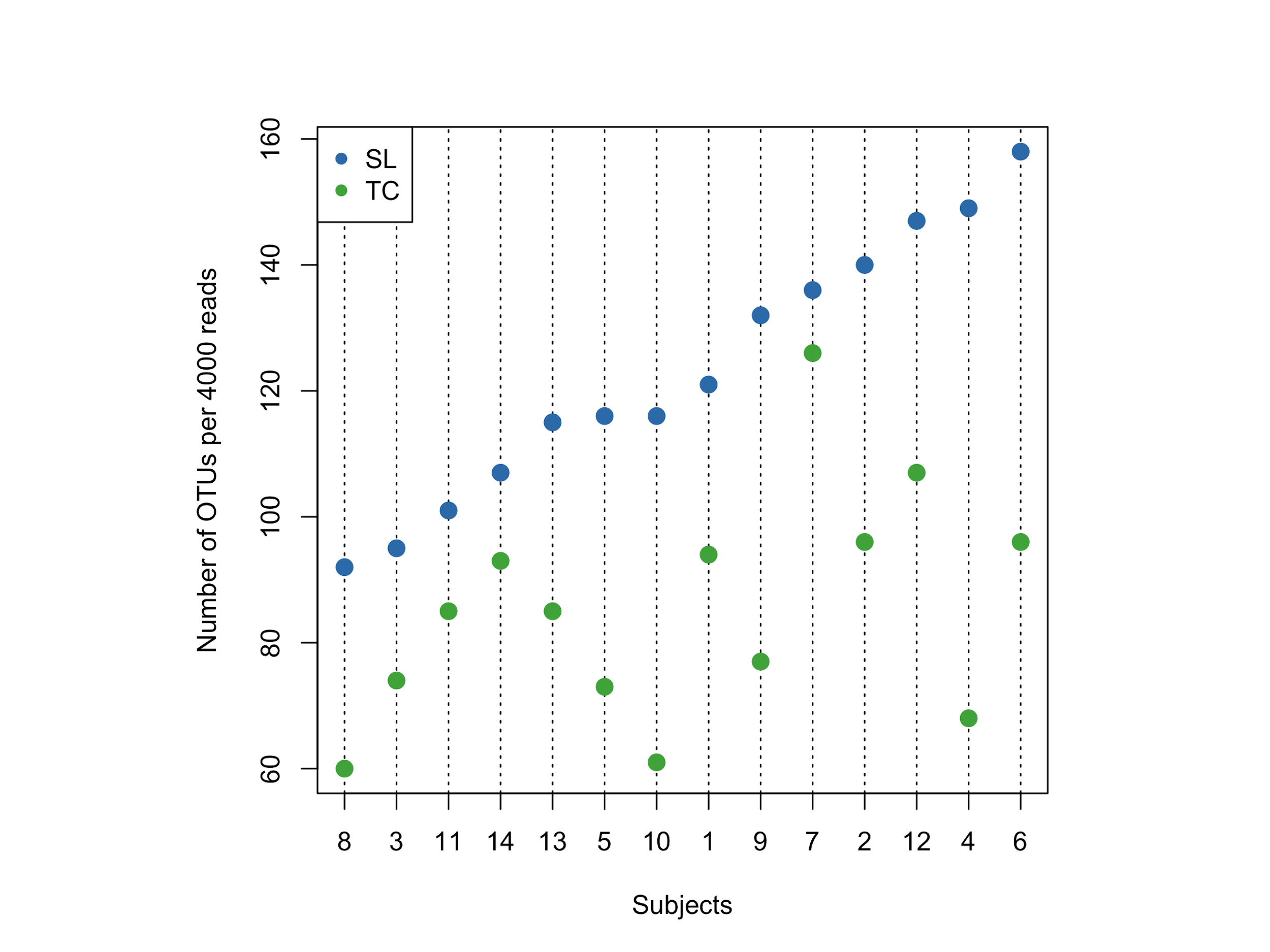

Supplement: S9 Fig — The number of OTUs is calculated following rarefaction to 4,000 reads per sample using R. (TIFF) [file pone.0174782.s009.tiff]
