# Supplementary material for: Relative abundance of total subgingival plaque-specific bacteria in salivary microbiota reflects the overall periodontal condition in patients with periodontitis
Source: PLoS One. 2017 Apr 3;12(4):e0174782. doi: 10.1371/journal.pone.0174782 (PMC5378373; doi:10.1371/journal.pone.0174782)
Supplement: S1 Table — BOP, bleeding on probing; PCR, plaque control record. (DOCX) [file pone.0174782.s010.docx]

**S1 Table**. Clinical condition of each subject in this study.

|  |  |  |  | Pre-therapy | | | | |  | Post-therapy | | | | |  | Supportive therapy | | |
| --- | --- | --- | --- | --- | --- | --- | --- | --- | --- | --- | --- | --- | --- | --- | --- | --- | --- | --- |
| Subjects | Age | Sex | Number of teeth | Percentage of sites (%) | | | PCR (%) | BOP (%) |  | Percentage of sites (%) | | | PCR (%) | BOP (%) |  | Number of revisiting | Interval (months) | The interval just before the 2nd sampling (months) |
|  |  |  |  | ≤3 mm | ≥4 mm | ≥7 mm |  |  |  | ≤3 mm | ≥4 mm | ≥7 mm |  |  |  |  |  |  |
| 1 | 46 | Male | 27 | 22.3 | 77.7 | 29.6 | 90.7 | 89.5 |  | 76.5 | 23.5 | 3.1 | 36.1 | 46.9 |  | 4 | 3-5 | 3 |
| 2 | 58 | Male | 22 | 78.0 | 22.0 | 0.8 | 80.7 | 63.6 |  | 91.7 | 8.3 | 1.5 | 68.2 | 34.1 |  | 6 | 3-4 | 4 |
| 3 | 73 | Male | 23 | 58.7 | 41.3 | 1.4 | 67.4 | 23.9 |  | 86.3 | 13.7 | 1.4 | 30.4 | 12.3 |  | 3 | 3 | 3 |
| 4 | 64 | Male | 28 | 64.3 | 35.7 | 1.8 | 82.1 | 57.7 |  | 76.4 | 23.6 | 0 | 83.3 | 45.7 |  | 2 | 3-4 | 3 |
| 5 | 58 | Male | 28 | 30.9 | 69.1 | 16.7 | 67.0 | 73.8 |  | 66.7 | 33.3 | 1.8 | 56.3 | 59.5 |  | 4 | 4-5 | 5 |
| 6 | 51 | Female | 25 | 27.3 | 72.7 | 12.7 | 93.0 | 100 |  | 91.6 | 8.4 | 1.3 | 38.5 | 27.6 |  | 2 | 2-3 | 3 |
| 7 | 57 | Female | 27 | 58.7 | 41.3 | 0.6 | 72.2 | 74.1 |  | 96.9 | 3.1 | 0 | 33.3 | 18.5 |  | 4 | 4-5 | 5 |
| 8 | 35 | Male | 19 | 71.9 | 28.1 | 1.8 | 59.2 | 50.9 |  | 82.4 | 17.6 | 0 | 51.3 | 39.5 |  | 7 | 3-4 | 3 |
| 9 | 72 | Male | 27 | 68.5 | 31.5 | 0 | 57.4 | 34.6 |  | 84.0 | 16.0 | 0 | 64.8 | 37.0 |  | 2 | 4 | 4 |
| 10 | 48 | Female | 30 | 60.6 | 39.4 | 6.1 | 72.5 | 67.2 |  | 84.5 | 15.5 | 1.1 | 35.0 | 30.0 |  | 4 | 3-4 | 4 |
| 11 | 59 | Male | 21 | 78.6 | 21.4 | 7.1 | 66.7 | 54.0 |  | 90.4 | 9.6 | 5.6 | 31.0 | 31.7 |  | 5 | 4 | 4 |
| 12 | 63 | Male | 20 | 14.2 | 85.8 | 33.3 | 67.5 | 80.0 |  | 39.1 | 60.9 | 11.7 | 50.0 | 55.8 |  | 7 | 1-4 | 3 |
| 13 | 43 | Female | 25 | 50.7 | 49.3 | 13.3 | 84.0 | 90.0 |  | 83.3 | 16.7 | 2.8 | 36.5 | 38.2 |  | 2 | 3 | 3 |
| 14 | 59 | Female | 27 | 61.8 | 38.2 | 11.7 | 73.1 | 54.9 |  | 78.2 | 21.8 | 5.8 | 37.5 | 38.5 |  | 1 | 4 | 4 |
